# Numerical Simulation for Thermal Shock Resistance of Thermal Protection Materials Considering Different Operating Environments

**DOI:** 10.1155/2013/324186

**Published:** 2013-08-01

**Authors:** Weiguo Li, Dingyu Li, Ruzhuan Wang, Daining Fang

**Affiliations:** ^1^College of Resource and Environment Science, Chongqing University, Chongqing 400030, China; ^2^LTCS and College of Engineering, Peking University, Beijing 100871, China

## Abstract

Based on the sensitivities of material properties to temperature and the complexity of service environment of thermal protection system on the spacecraft, ultrahigh-temperature ceramics (UHTCs), which are used as thermal protection materials, cannot simply consider thermal shock resistance (TSR) of the material its own but need to take the external constraint conditions and the thermal environment into full account. With the thermal shock numerical simulation on hafnium diboride (HfB_2_), a detailed study of the effects of the different external constraints and thermal environments on the TSR of UHTCs had been made. The influences of different initial temperatures, constraint strengths, and temperature change rates on the TSR of UHTCs are discussed. This study can provide a more intuitively visual understanding of the evolution of the TSR of UHTCs during actual operation conditions.

## 1. Introduction

Ultrahigh-temperature ceramics (UHTCs) is a family of materials that have melting points higher than 3000°C and can be potentially used at temperatures above 2000°C in an oxidizing environment. As important ceramics and promising candidates for high temperature applications of thermal protection systems (TPS), UHTCs are attracting growing attention. However, currently, due to lack of sufficient knowledge on UHTC thermal protection materials, development and application of these materials have been limited.

Current research shows that TSR of ceramic materials is poor because of their inherent brittleness. Thermal shock is the cause of damage of ceramic materials [1, 2]. The TSR performance of ceramic materials depends on the mechanical properties and thermal properties of the material. Moreover, the effects of the geometry of components and environmental media are also very important points [3] because the properties of the materials are sensitive to the temperatures and the complex thermal environments experienced by the spacecraft surface. At present, the research of TSR mostly focuses on the effects of surface defects and indentation crack length [3] and the influences of crack density [2, 4], particle reinforced [5, 6] or whisker reinforced [7], glass phase [8] on TSR performance in experimental way. Theoretical research of the effect of surface heat transfer coefficient on TSR has been made [9], and several evaluation theories of TSR has been reported [3, 10, 11]. Moreover, Song et al. enhanced thermal shock resistance of ceramics through biomimetically inspired nanofins which had proved to be a very effective way [12]. However, few experiments have considered the influence of temperature of the cooling medium and the different external constraint conditions because it is difficult to conduct. And the current experiment is difficult to simulate the thermal environment and external constraint conditions suffered by the UHTCs in the actual operation and difficult to reveal the changes of the TSR of the thermal protection materials in the operating process. Moreover, such experiments cannot meet the demand of comprehensively understanding the TSR of materials.

Therefore, hafnium diboride (HfB_2_) is used to study the effects of the external constraint conditions and different thermal environment on the TSR of the UHTC in detail through numerical simulation, due to the restrictions of current experiments and the lack of theories. The study provides a more comprehensive understanding of the changes of TSR in the course of the entire service of the material. Furthermore, this paper provides some possible ways for the application design, improving the TSR and reliability of the thermal protection materials.

## 2. Finite Element Analysis Model

**Figure 1:**
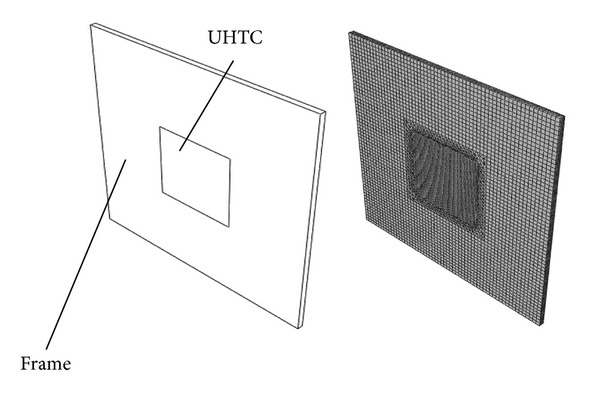
Geometric model and the finite element mesh.

The large scale general finite element analysis software ABAQUS was used for the analysis. In order to simulate the effects of different external constraint conditions on the TSR performance of the UHTC thermal protection materials, the geometric model and the finite element mesh are employed, as shown in Figure 1. Assumed that (1) the UHTC material is isotropic; (2) there is no initial stress in the material at thermal shock initial temperature; (3) the connection between the UHTC material and the external constraints (the frame) is perfect; (4) the parameters of the thermal physical properties of the UHTC material are functions of temperature; (5) The temperature of the external constraint (the frame) is constant.

Hafnium diboride (HfB_2_) is used for simulation example, with material properties [13–15] shown in Table 1. According to calculation requirements, various Young's modulus values of the frame (from 0 to 650 GPa) are selected to simulate the different constraint strength of external constraints applied on the UHTC plate. The Poisson's ratio of the frame material is taken to be 0.3.

## 3. Results and Discussion

Tensile stress is formed at the surface of ceramic materials during the cooling process, which is more dangerous than the heating process. Therefore, this paper takes the calculation of cooling process as a model for analysis. In the calculations, when the UHTC plate surface maximum tensile stress reaches the strength of materials corresponding to the current temperature (i.e., the materials are damaged, and the surface temperature is the critical fracture temperature). The difference between the critical fracture temperature and thermal shock initial temperature (*T*) is the critical fracture temperature difference Δ*T*. The temperature-dependent strength of the UHTC material can be calculated as follows [16]:

(1) σth(T)=[(σth0)2E0E(T)  ×[1−1∫0TmCp(T)dT∫0TCp(T)dT]]1/2,

where *σ*_th_(*T*) is the temperature-dependent fracture strength, *σ*_th_^0^ is the fracture strength at the reference temperature, *E*_0_ is Young's modulus at the reference temperature of the material, and *E*(*T*) is the temperature-dependent Young's modulus.

 Single-sided cooling of the UHTC plate in various cooling rates with different constraint conditions (corresponding to different frame material Young's modulus) and thermal shock initial temperatures are also simulated. 

**Figure 2:**
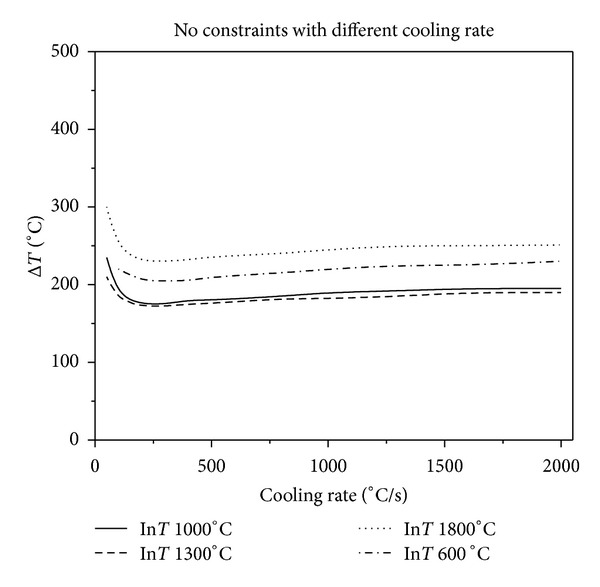
Relationship between critical fracture temperature Δ*T* corresponding to different thermal shock initial temperature (in the figure is InT, the same as follows) and cooling rate without constraints.

**Figure 3:**
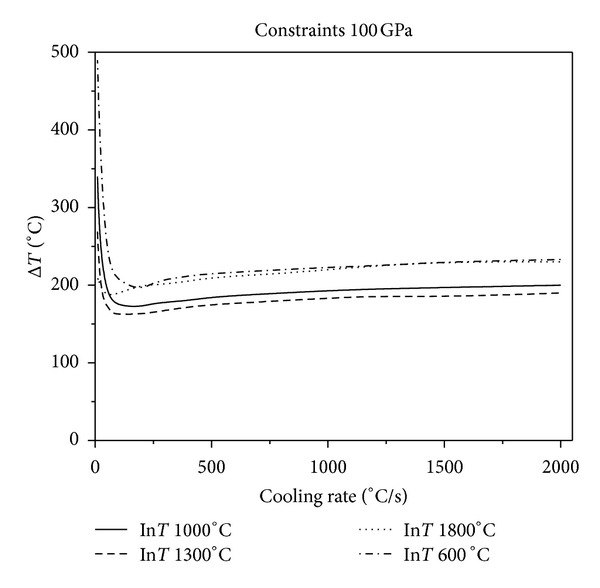
Relationship between critical fracture temperature Δ*T* corresponding to different thermal shock initial temperature and cooling rate which Young's modulus of constraint material is 100 GPa.

**Figure 4:**
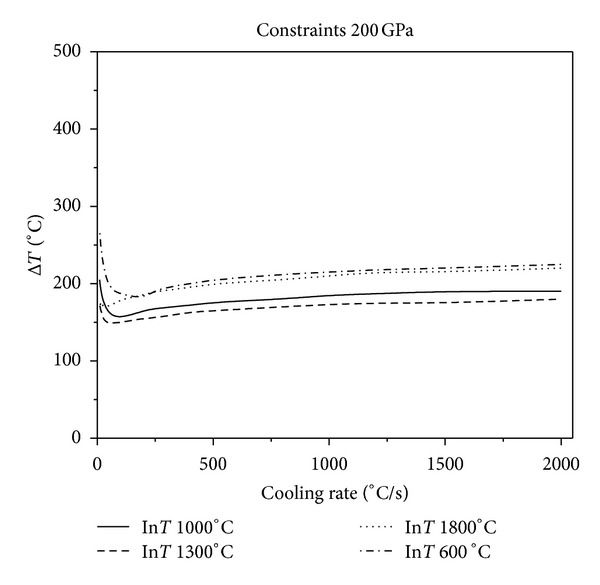
Relationship between critical fracture temperature Δ*T* corresponding to different thermal shock initial temperature and cooling rate which Young's modulus of constraint material is 200 GPa.

**Figure 5:**
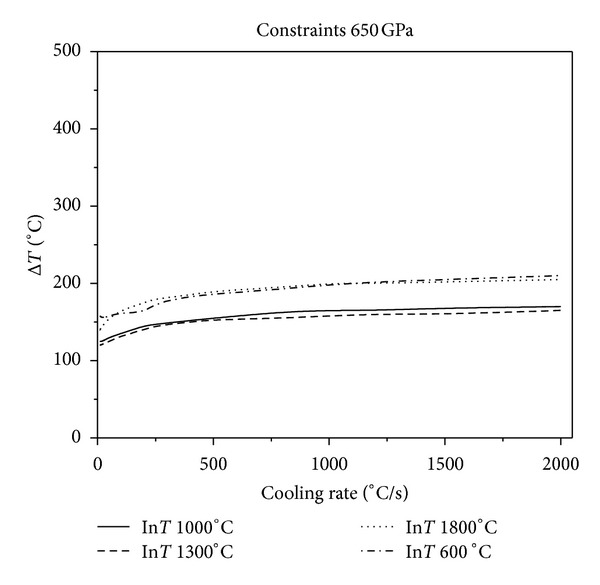
Relationship between critical fracture temperature Δ*T* corresponding to different thermal shock initial temperature and cooling rate which Young's modulus of constraint material is 650 GPa.

Figures 2, 3, and 4 show that, in the same constraint, the critical fracture temperature difference Δ*T* that UHTCs can withstand rapidly decreases initially and slowly rises as the cooling rate increases. Therefore, there is a dangerous zone of cooling rate where the thermal shock resistance is lowest when the cooling rate is relatively minor. From this result, the conclusions, that the higher the cooling rate the smaller the critical temperature difference that the material can withstand, which are obtained from experiments in previous literature is considered one sided. This is due to restrictions of experimental methods. Experiments cannot reproduce the whole real and complex processes of thermal shock that the thermal protection materials suffer during the causative processes. The common experimental methods are so simple and harsh that the understanding of TSR of materials is extremely one sided. This is especially true for the UHTC thermal protection materials, which suffer from a wide and drastic temperature change. Simulating and studying the impact that various operating environments impose on TSR is therefore crucial. In addition, the figures show that the higher the thermal shock initial temperature, the lower the cooling rate corresponding to the minimum critical temperature of different initial temperature. Under the same circumstances, with the enhancement of external constraints, the temperature difference that materials can withstand decreases and the lowest temperature point comes close to the vertical axis. When constraints are enhanced to a certain extent, as Figure 5, there is no visible lowest point in the temperature curve; however, with the increase of cooling rate, the temperature curve increases slowly and tends to be constant.

**Figure 6:**
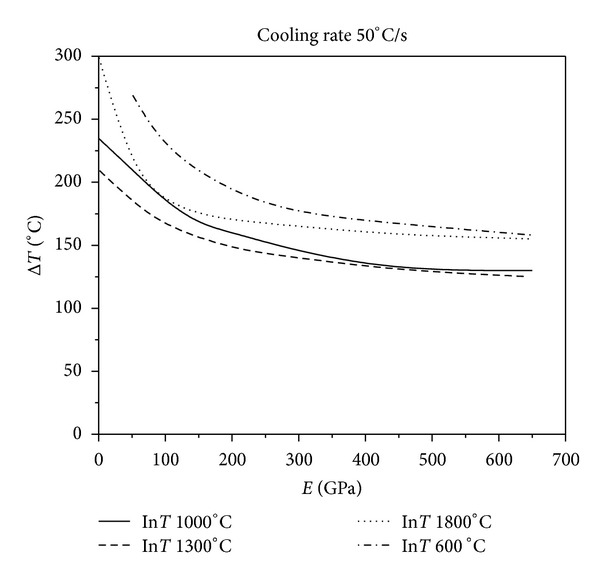
Relationship between critical fracture temperature corresponding to different thermal shock initial temperature and Young's modulus of constraint material under the cooling rate 50°C/s.

**Figure 7:**
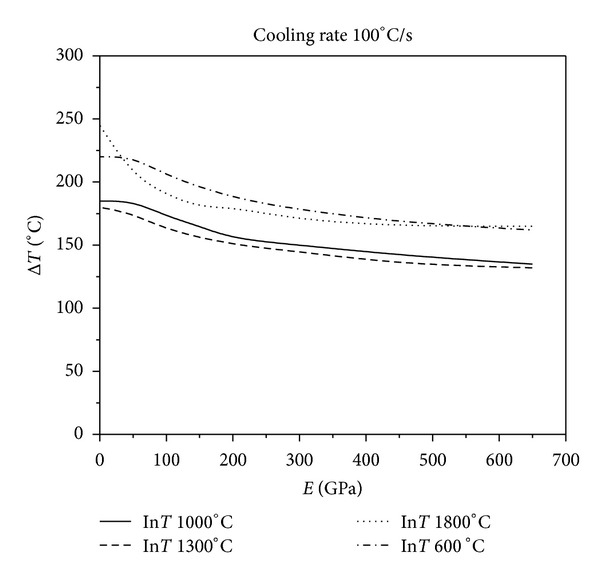
Relationship between critical fracture temperatures corresponding to different thermal shock initial temperature and Young's modulus of constraint material under the cooling rate 100°C/s.

**Figure 8:**
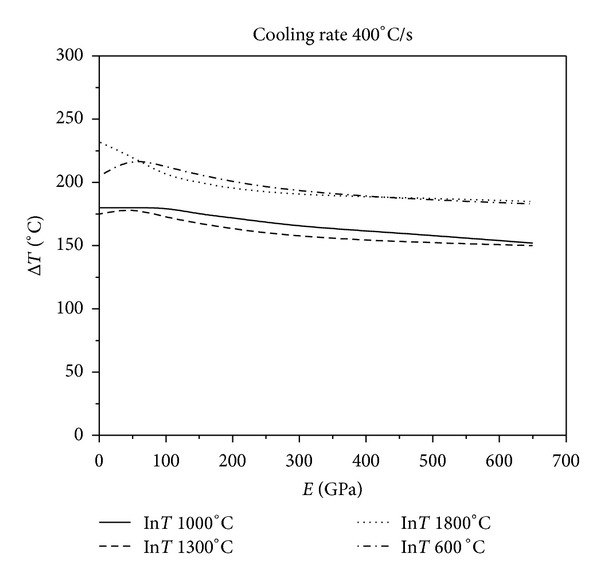
Relationship between critical fracture temperatures corresponding to different thermal shock initial temperature and Young's modulus of constraint material under the cooling rate 400°C/s.

**Figure 9:**
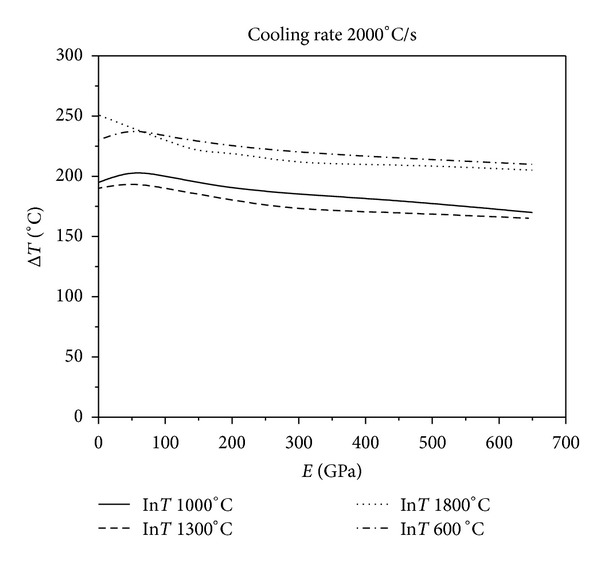
Relationship between critical fracture temperatures corresponding to different thermal shock initial temperature and Young's modulus of constraint material under the cooling rate 2000°C/s.

Figures 6 to 9 show the results of various levels of constraints for different initial temperatures at four different cooling rates. As Figure 6 shows, when the cooling rate is 50°C/s, the critical fracture temperature difference of the material corresponding to the same initial temperature of thermal shock decreases monotonically as the external constraints increase. Figures 7, 8, and 9 show that the critical fracture temperature difference of the material initially increases and then decreases with the enhancement of external constraints when the cooling rate is higher than a certain level. This case can be seen when the initial temperatures of thermal shock are 600, 1000, and 1300°C. However, when the initial temperature of thermal shock is 1800°C, the critical fracture temperature difference of the material decreases monotonically with the increase of external constraints. These figures also show that when the initial temperature is 1300°C, the critical fracture temperature difference is the smallest. This conclusion again proves that there is a danger zone corresponding to the initial temperature of thermal shock. Moreover, if some certain constraints are applied on the UHTC materials, the materials can withstand higher critical fracture temperature difference. And the critical fracture temperature difference is sensitive to external constraints when constraints level is small; however, with the increase of external constraints, the sensitivity decreases rapidly. The lower the cooling rate, the more sensitive the critical fracture temperature difference is to the changes of external constraints. From these results, it can be seen that the reasonable adjustments to the connections between the thermal protection materials and the main structure in the corresponding causative environment can significantly improve the TSR of the thermal protection materials.

**Figure 10:**
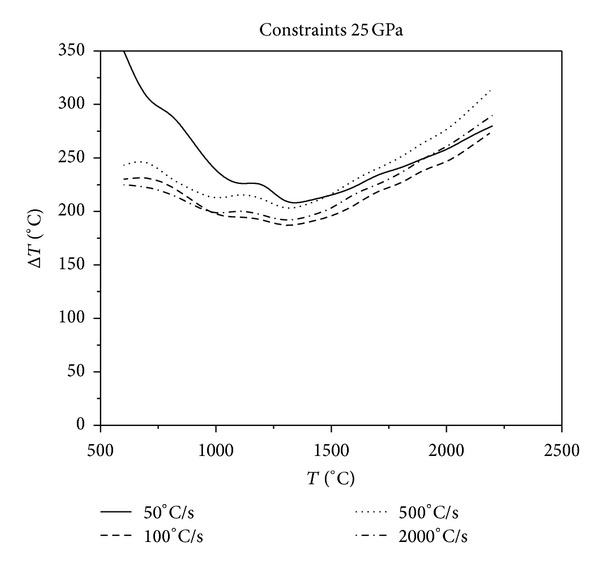
Relationship between critical fracture temperatures corresponding to different cooling rates and thermal shock initial temperatures under the constraints of 25 GPa.

**Figure 11:**
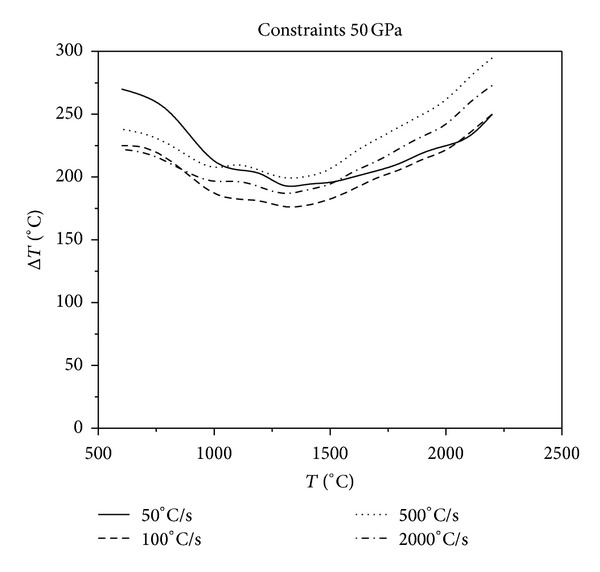
Relationship between critical fracture temperatures corresponding to different cooling rates and thermal shock initial temperatures under the constraints of 50 GPa.

**Figure 12:**
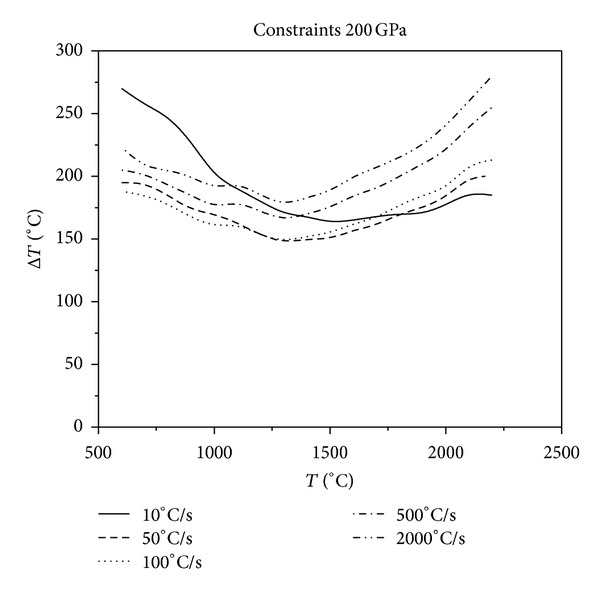
Relationship between critical fracture temperatures corresponding to different cooling rates and thermal shock initial temperatures under the constraints of 200 GPa.

**Figure 13:**
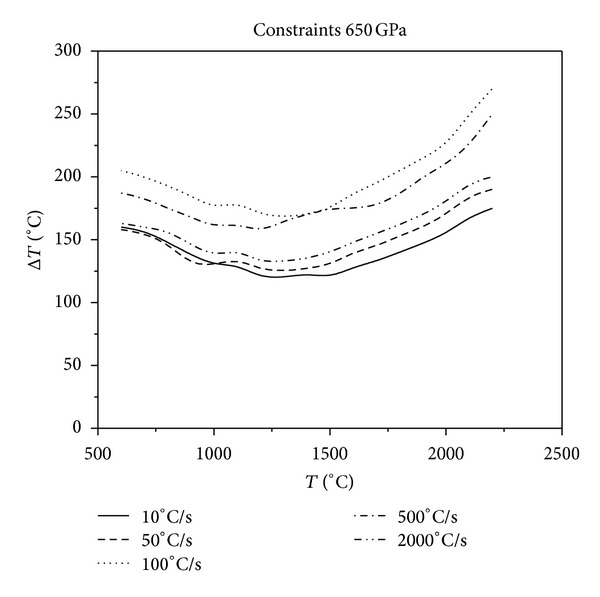
Relationship between critical fracture temperatures corresponding to different cooling rates and thermal shock initial temperatures under the constraints of 650 GPa.

As it can be seen in Figures 10, 11, 12, and 13, with the same constraints, the critical fracture temperature difference decreases initially and then increases at a later point as the initial temperature of thermal shock increases. Therefore, there is a dangerous district of the initial temperature of thermal shock where the TSR is lowest. When the initial temperature of thermal shock is lower (approximately 1300°C), the lower the cooling rate, the greater the critical fracture temperature difference. Whereas, when the initial temperature of thermal shock is higher, the higher the cooling rate, the greater the critical fracture temperature difference. In addition, in the role of external constraints, when the cooling rate reaches a certain value, the higher the cooling rate, the greater the critical fracture temperature difference. This conclusion is opposite to that reported in the literature [17] that the “higher the cooling rate, the smaller the critical fracture temperature difference.” Using the common experimental methods that do not consider the initial temperature of thermal shock, cooling medium temperature and external constraints to study TSR of materials would be one sided. The evolution of TSR in the course of the causative is comprehensively poorly understood, especially for UHTC thermal protection materials. Thus, the research of the impact that thermal environment and external constraints imposed on TSR is crucial.

## 4. Conclusions

A reasonable finite element model was established by considering the factors of external constraints and thermal environment in this paper. The result shows that when considering the external constraints and the thermal environment, the TSR of UHTC thermal protection materials can no longer take into account only the TSR of the material its own. 

There is a dangerous zone of cooling rate where the TSR is lowest when certain constraints are applied upon the material. The critical fracture temperature difference is sensitive to external constraints when the constraints are small; however, with the increase of the external constraints, the sensitivity decreases rapidly. In addition, in the role of external constraints, when the temperature change rate reaches a certain value, the higher the temperature change rate, the greater the critical fracture temperature difference. That is to say reasonable adjustments in the connections between the thermal protection materials and the main structures can significantly improve the TSR of thermal protection materials, which provides new ideas for structural optimization. 

## Figures and Tables

**Table 1:** Temperature-dependent material properties of HfB_2_.

Material parameters	Values and expressions
*T*_*m*_ (°C)	3400
*E* (GPa)	*E* = *E*_0_ − *BTe* ^−*T*_*m*_/*T*^ + *B*_1_(*T* − *B*_2_ *T*_*m*_ + |*T* − *B*_2_ *T*_*m*_|)*e* ^−*T*_*m*_/*T*^
*E*_0_, *B*_0_, *B*_1_, *B*_2_	441, 2.54, 1.9, 0.363
*α* (°C^−1^)	(2ln(*T*) − 5) × 10^−6^
*k* (W·(m·°C)^−1^)	– 8.3455 × ln(*T*) + 127.68
*ν*	0.12
*C*_*p*_(*T*) (cal/mol)	73.346 + 7.824 × 10^−3^ *T* − 2.301 × 10^6^ *T* ^2^
*ρ* (g·cm^−3^)	10.5
